# Biomimetics of the pulmonary environment *in vitro*: A microfluidics perspective

**DOI:** 10.1063/1.5023034

**Published:** 2018-05-29

**Authors:** Janna Tenenbaum-Katan, Arbel Artzy-Schnirman, Rami Fishler, Netanel Korin, Josué Sznitman

**Affiliations:** Department of Biomedical Engineering, Technion–Israel Institute of Technology, 32000 Haifa, Israel

## Abstract

The entire luminal surface of the lungs is populated with a complex yet confluent, uninterrupted airway epithelium in conjunction with an extracellular liquid lining layer that creates the air-liquid interface (ALI), a critical feature of healthy lungs. Motivated by lung disease modelling, cytotoxicity studies, and drug delivery assessments amongst other, *in vitro* setups have been traditionally conducted using macroscopic cultures of isolated airway cells under submerged conditions or instead using transwell inserts with permeable membranes to model the ALI architecture. Yet, such strategies continue to fall short of delivering a sufficiently realistic physiological *in vitro* airway environment that cohesively integrates at true-scale three essential pillars: morphological constraints (i.e., airway anatomy), physiological conditions (e.g., respiratory airflows), and biological functionality (e.g., cellular makeup). With the advent of microfluidic *lung-on-chips*, there have been tremendous efforts towards designing biomimetic airway models of the epithelial barrier, including the ALI, and leveraging such *in vitro* scaffolds as a gateway for pulmonary disease modelling and drug screening assays. Here, we review *in vitro* platforms mimicking the pulmonary environment and identify ongoing challenges in reconstituting accurate biological airway barriers that still widely prevent microfluidic systems from delivering mainstream assays for the end-user, as compared to macroscale *in vitro* cell cultures. We further discuss existing hurdles in scaling up current *lung-on-chip* designs, from single airway models to more physiologically realistic airway environments that are anticipated to deliver increasingly meaningful whole-organ functions, with an outlook on translational and precision medicine.

## I. INTRODUCTION

In recent years, microfluidics have gained significant momentum in lying the foundations for constructing *in vitro* models that mimic physiologically relevant organ functions, a field increasingly known as *body-* or *organ-on-chips.*^1–3^ Despite impressive advancements and an expanding pallet of available designs,^4,5^ conceiving *in vitro* microfluidic models of the pulmonary system that are robust and biologically faithful still remains a great technological challenge for advancing both fundamental and translational research in respiratory physiology. Notably, many of the hurdles faced in recreating biomimetic *in vitro* analogues of the pulmonary environment at the microscale are exemplified when addressing the gas-exchange region of the lungs.

With the body's largest surface area directly exposed to the external environment^6^ (i.e., nearly 100 m^2^ in an average adult), the lungs' principal function represents not only a cornerstone of human life, guaranteeing foremost oxygen and carbon dioxide exchange, but also a zone susceptible to insult and injury^7–9^ as a result of chronic or acute exposure (e.g., environmental or anthropogenic particular matter including nanoparticles, airborne viruses, or bacteria, etc.). Concurrently, the accessibility to a vast tissue surface offers a potent non-invasive gateway for inhalation therapeutics in the context of both topical^10,11^ (e.g., antibiotics and chemotherapy) and systemic delivery^12–14^ (e.g., gene therapy and vaccination). Alongside, *in vitro* microfluidic platforms of the respiratory airways are undergoing rapid development motivated amongst others by lung disease modeling,^15–20^ cytotoxicity studies,^16,21^ cancer diagnosis,^22^ and drug screening assays.^20,23–25^ Among the arguments advocated for perseverance lies, for example, the prospect of alternatives to animal testing^21,26,27^ both as a testing platform for the above-mentioned applications as well as for individualized disease models, allowing an optimized match between patient and treatment.^18^ Yet, the state of the field is anticipated to still be in its infancy. Ongoing research efforts are attempting to overcome many of the outstanding hurdles towards integrating (i) anatomical and morphological realism, (ii) physiological respiratory flow conditions, and (iii) perhaps most importantly biological functionality into attractive *lung-on-chip* models; a term we loosely define here as any microfluidic platform that mimics one or multiple features of the respiratory organ.

In this article, we review and discuss ongoing developments in the field of *in vitro* models of the pulmonary environment, with a special emphasis on microfluidic platforms mimicking respiratory airways and the gas-exchange region. We first address three broadly identified areas (i.e., anatomical constraints, physiological conditions, and biological functionality) that may be regarded as the underlying pillars for devising biomimetic pulmonary models. We revisit traditional macroscale *in vitro* models replicating the air-liquid interface (ALI) and the persevering efforts undergone to miniaturize such platforms and integrate true-scale features of the airway barrier within microfluidic systems. Importantly, we emphasize some of the existing challenges in scaling-up current micro-technologies, from single airway models to more realistic airway networks that strive to better capture whole-organ functions. Altogether, existing and future endeavours are anticipated to advance such micro-platforms in becoming more mainstream assays at the hands of the end-user, compared to traditional macroscale *in vitro* cell cultures.

## II. TRANSLATING THE PULMONARY ANATOMY FOR MICROFLUIDICS

**FIG. 1.**
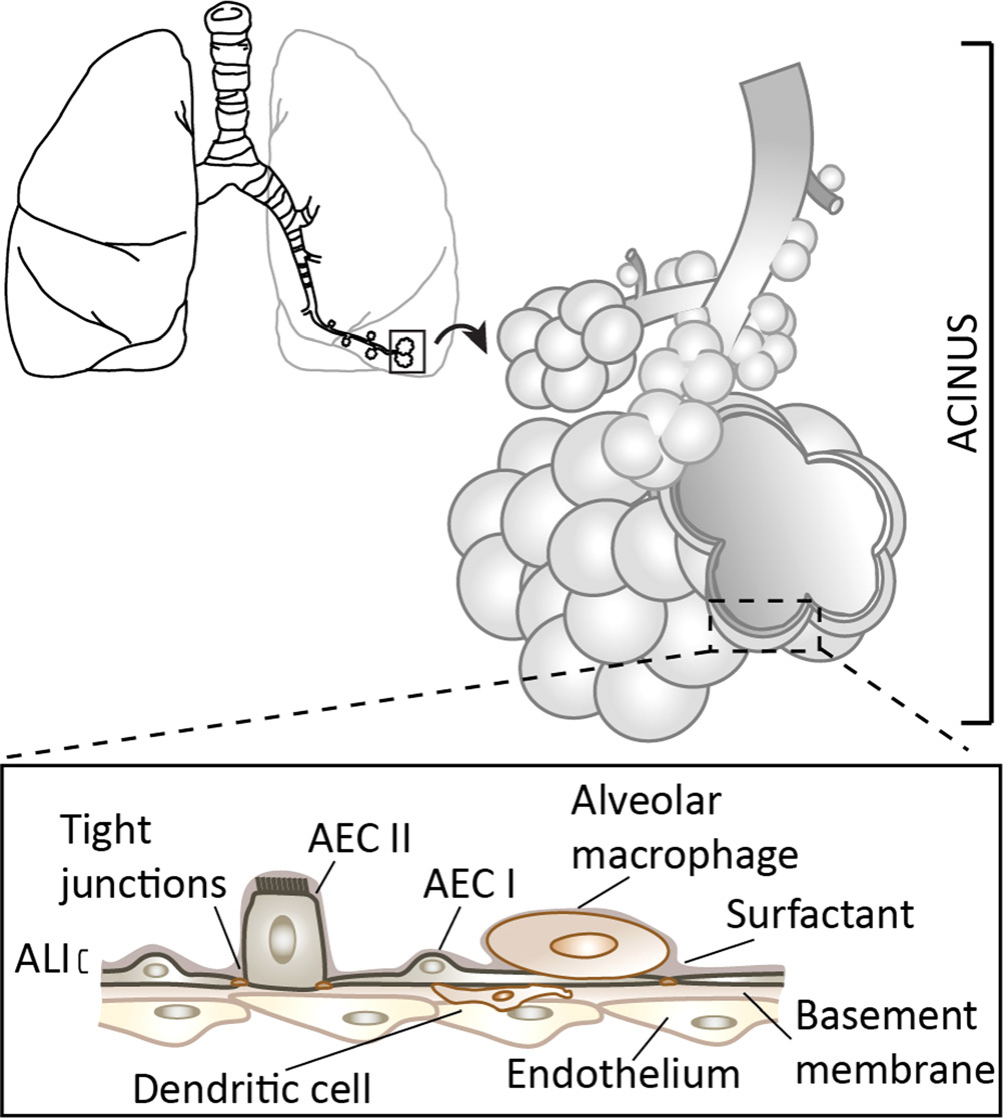
Schematic of the branching structure of the lungs and the gas-exchange region (i.e., pulmonary acinus). Inset: Schematic of the alveolar septal wall structure and its cellular make-up separating the air- (apical side) from the blood-phase (basal side). A confluent uninterrupted epithelial sheet consisting of alveolar epithelial cells AEC types I and II coats the alveolar wall, where a thin liquid layer lies upon the cellular lining, creating the so-called air-liquid interface (ALI). Tight junctions maintain the integrity of the epithelial monolayer. Airway macrophages, along with dendritic cells, constitute the underlying components of the barrier that prevent amongst other foreign matter from translocating across the blood-side (i.e., endothelium).

The lungs' vast airspace is arranged within a bifurcating tree structure spanning ∼20 airway generations, where length scales cover several orders of magnitude, i.e., from the trachea (∼1 cm) down to the alveoli (∼100 *μ*m). While the conductive region (also known as the “dead space”) does not participate in gas exchange, the pulmonary acinar region is designed for efficient oxygen and carbon-dioxide transfer into blood and accounts for >90% of total lung volume.^28^ Anatomically, the pulmonary acinus is defined as the branched complex of sub-millimetre alveolated airways^29^ (Fig. 1), with alveoli first appearing past the 15th generation or so. Estimates typically place the total number of acini at over 30 000 in an average adult human lung; each acinus holds ∼200 mm^3^ with ∼10 000 alveoli arranged across ∼6 to 12 generations.^29^ Due to the quasi-dichotomous branching nature of the pulmonary tree,^30^ approximately half of the pulmonary acinar volume is located in the lungs' last generation. Together, the body holds nearly half a billion alveoli, where the mean volume of an alveolus^31^ is approximately 4 × 10^−6^ *μ*m^3^ with a characteristic length scale of ∼100–200 *μ*m.

**FIG. 2.**
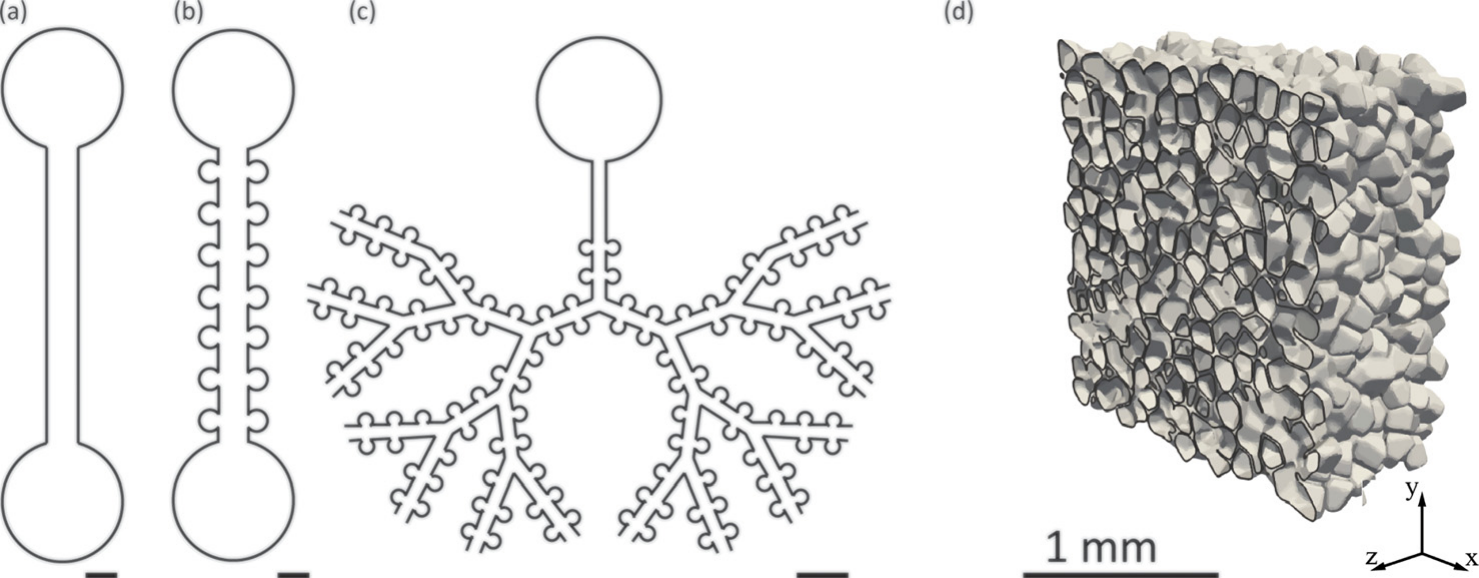
Schematic examples (a)–(c) of microfluidic embodiments mimicking the pulmonary airway environment. Designs range from (a) simple, straight channels to (b) alveolated channels lined with regularly positioned alveolar (cylindrical) cavities and (c) bifurcating tree networks of alveolated airways spanning multiple generations. (a)–(c) Scale bar corresponds to 400 *μ*m. While sample device layouts shown in (a)–(c) are limited to 2D planar designs due to standard microfabrication constraints, the innate environment in the lung depths is more faithfully captured by (d) heterogeneous space-filling pulmonary acinar structures as shown in this computer-aided design (CAD) rendering (adapted from Ref. 156). An arbitrary 2D cut-plane through the 3D space highlights alveolar cavities that form a tightly packed sleeve around acinar ducts where adjacent alveoli are separated by dividing membranes (i.e., inter-alveolar septa). Reproduced with permission from Eur. J. Pharm. Sci. **113**, 53–63 (2018).^156^ Copyright 2018 Elsevier.

Topologically, alveolar cavities surround acinar ducts and form a tightly packed sleeve where adjacent alveoli are separated by dividing membranes (i.e., inter-alveolar septa). While the shapes of alveoli are intrinsically heterogeneous, resembling spheroids, ellipsoids, and cylindroids,^32^ the gas-exchange region may be somewhat generalized as analogous to honeycomb structures comprising hollow polyhedral-shaped cavities.^33^ Here, microfabrication techniques (e.g., photo-lithography, thin-film deposition, etching, etc.) generally have the ability to capture at true scale several of the relevant morphological features and dimensions of the pulmonary airspace. Yet, the 3D space-filling nature of the gas-exchange region remains prohibitively complex to recreate *in vitro* when considering standard microfluidic techniques,^34,35^ as microfabricated patterns are typically restricted to two-dimensional (2D) planar geometries [Figs. 2(a)–2(c)]. Not surprisingly, existing microfluidic airway models have most often relied on simple geometries consisting of straight rectangular channels^15,23,24,36,37^ [Fig. 2(a)] or well-shaped geometries that mimic isolated alveolar sacs.^38^

More recently, models of alveolated channels^39,40^ [Fig. 2(b)] and alveolated airway trees spanning several bifurcating generations^41–43^ [Fig. 2(c)] have begun to surface in an effort to replicate more faithfully the innate acinar morphology. While the general dimensions of these devices are somewhat in good agreement with lung morphometry,^29^ microfabrication constraints represent nonetheless a limiting factor. To begin, both acinar ducts and alveoli lie within a single plane. Hence, the 3D space-filling structure of the tree, reminiscent of a spongious environment [Fig. 2(d)], is entirely omitted. Instead, planar 2D branching angles are most often selected to maximize the number of bifurcating generations but may not necessarily reflect anatomical features. Importantly, microfluidic airways have been restricted to acinar duct models exhibiting large inter-spacing between alveolar cavities [Figs. 2(b) and 2(c)], compared with an innate environment where alveoli share inter-alveolar septa [Fig. 2(d)]. This discrepancy yields a low ratio of alveolar-to-ductal volume;^44^ a limitation not necessarily critical in replicating the biological functions of the epithelial barrier and air-liquid interface (see Sec. IV) but important in evaluating realistic aerosol deposition patterns.^41^ This latter point may be pertinent for cytotoxicity and dosimetry, or alternatively, in targeting therapeutic delivery.^45,46^

Beyond the aforementioned *in vitro* airway microsystems, advances in microfabrication techniques have helped deliver 3D micro-engineered environments with the advent of 3D printing, additive manufacturing, and various stamping techniques.^47–50^ On the one hand, such progress has arisen from the desire to devise more functional *organ-on-chips.*^51^ For example, mimicking 3D networks of the microvasculature has received growing attention: efforts to recreate *in vitro* microcirculatory architectures have been motivated by tissue engineering applications as well as vasculo- and angiogenesis studies.^52–55^ In parallel, recreating microvascular networks using microfluidics has also inspired novel artificial lung designs and blood oxygenators, as recently reviewed,^56^ despite ongoing challenges in meeting physiological *in vivo* requirements of gas exchange at the whole-organ level. Though some of the underlying 3D branching patterns and characteristic length scales (∼100 *μ*m and smaller) of the microvasculature are somewhat analogous to pulmonary airways, it is still unclear how closely the complexities of the lung morphology and the inherent three-dimensionality of the acinar airspace may be recreated [Fig. 2(d)]. Nevertheless, it is anticipated that state-of-the-art 3D printing, or alternative techniques, will be increasingly borrowed to conceive evermore faithful *in vitro* analogues of the airway environment, when replicating anatomy with high-fidelity is indeed critical.^41^

## III. ESTABLISHING RESPIRATORY FLOWS *IN VITRO*

Following birth, the lungs rapidly adapt into an air-filled environment. This configuration distinguishes respiratory airflows from traditional microfluidic setups driven by liquid-based applications.^57,58^ In what follows, we largely confine our discussion to the specifics of airflows in small and acinar airways; the characteristic lengths of such regions (see Sec. II) are indeed most suitable for microfluidic applications. We briefly recall that airflows in the conductive regions occur within considerably larger airways (i.e., ∼0.1–1 cm) and may be highly inertial if not transitional or turbulent (e.g., larynx and trachea); here, the reader is invited to consult detailed reviews on the topic.^59–61^ In contrast, airflows in deeper airways (e.g., bronchioles and beyond) embody small-scale flows in the realm of low-Reynolds-number fluid mechanics. Such flows may be briefly described as those giving rise to laminar flow patterns, including notably Poiseuille flow.^62,63^ Yet, research in the past two decades has helped elucidate how respiratory flows in the sub-millimetre regions of the lungs may diverge significantly from such simple descriptions,^64–67^ a consequence of two distinct yet coupled features of the gas-exchange region.

The first specificity revolves around the organ's anatomy which departs from cylindrical airways due to the structure of the acinar ducts [Sec. II, Fig. 2(d)] and the presence of alveolar cavities. Low-Reynolds-number cavity flows are generally known to exhibit a range of complex 3D flow patterns.^68,69^ Recent flow visualizations in microfluidic cylindrical cavities have exemplified the occurrence of various flow topologies under such flow regimes;^70^ depending on geometrical parameters (e.g., mouth opening angle, cavity depth, height-to-diameter ratio), patterns may include attached flow, a single recirculating zone, or multi-vortex flow structures. Hence, alveolar flows are anticipated to be intrinsically sensitive to the local airway morphology since alveolar walls determine ensuing boundary conditions (i.e., no slip at the wall). The second specificity lies in the transient nature of breathing due to the lungs' expansion and contraction motions which drive oscillatory pressure gradients: the acinar domain is in constant motion with relative volume changes that may reach well beyond 50% for a deep inhalation manoeuvre. Moreover, non-isotropic expansions of the parenchyma ensure that alveolar flow patterns are not quasi-steady but rather flow topologies may change constantly throughout the breathing cycle.^42,71–73^ Overall, the underlying parenchymal motion guarantees net convective exchange between inhaled and residual air.^74,75^

**FIG. 3.**
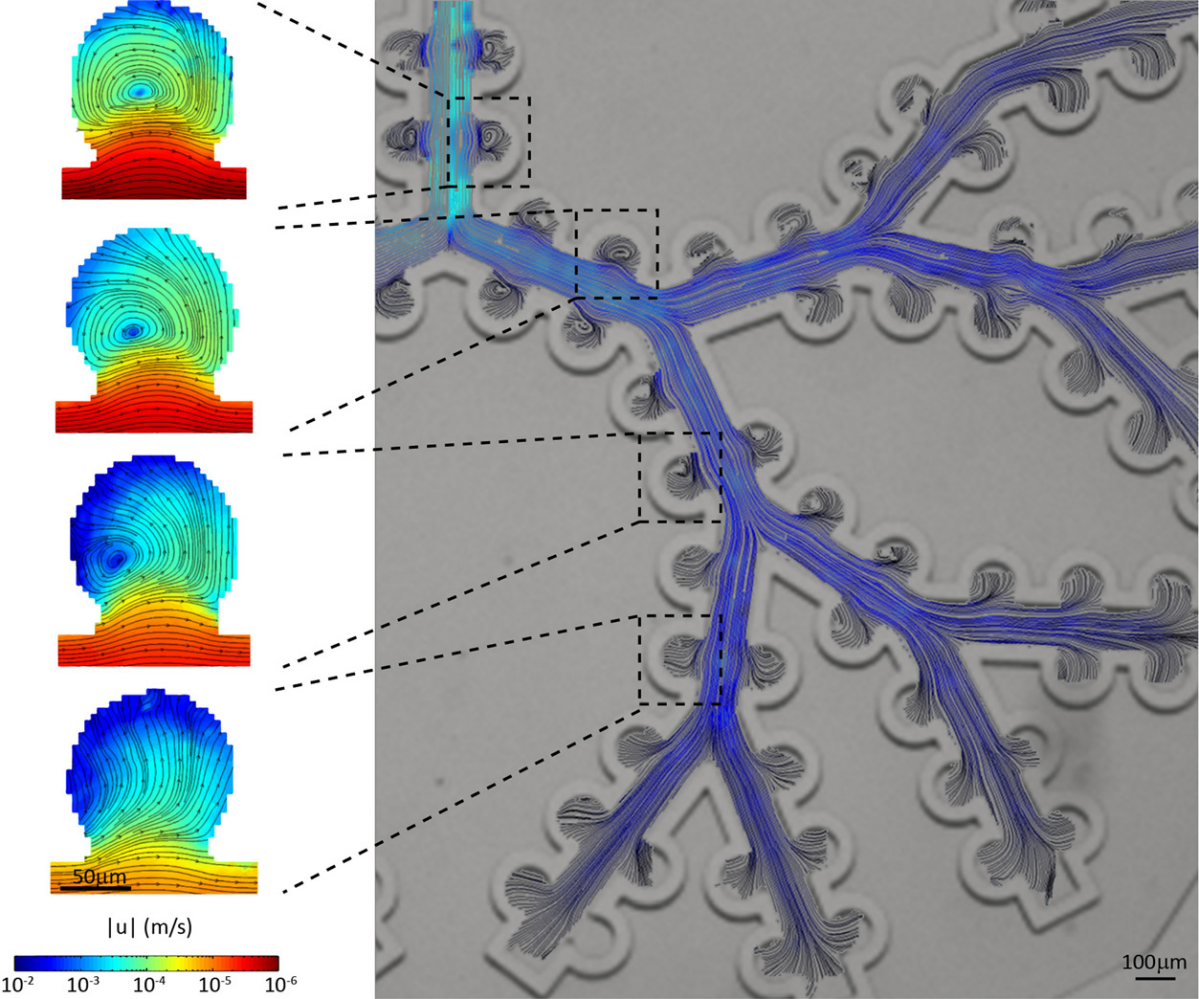
Quantitative flow visualizations of respiratory flow patterns characteristic of the pulmonary acinar meso- (tree) and microscale (alveoli) in a microfluidic alveolated tree model under moving wall conditions mimicking breathing motions (adapted from Ref. 41) While flows in the acinar ducts are reminiscent of smooth laminar channel flows, the particularity of the alveolar topology leads to the emergence of low-Reynolds-number cavity flows within, where flow patterns evolve as a function of location along the acinar tree, from recirculating (i.e., vortex) alveolar flows in proximal acinar generations to the presence of radial-like streamlines in more distal airways. Note that the field of view captures approximately half of the entire acinar tree structure, starting from the first generation (top left) down to the fifth generation. Reproduced with permission from Sci. Rep. **5**, 14071 (2015). Copyright 2015 Nature Publishing Group.

In considering microfluidic designs, respiratory flows obey the laws of mass conservation such that flow magnitudes in the acinar airways rapidly decrease as air is distributed at each bifurcation into deeper generations. In turn, the coupling between oscillatory shear flows in acinar ducts and the alveolar cavity openings leads to distinct flow configurations that are sensitive to the location of an alveolus along the acinar tree.^65,70^ Using micro-particle image velocimetry (*μ*PIV), measurements in microfluidic alveolated tree models^41,42^ have revealed the existence of a range of complex recirculating (i.e., vortex) flows in alveoli, with a gradual crossover to more radial-like streamlines in the deeper acinar generations (Fig. 3). Here, breathing motions that match physiologically realistic strains are mimicked using thin deformable polydimethylsiloxane (PDMS) walls separating acinar ducts and alveoli from surrounding water-filled chambers.^43^

Despite limitations in the anatomical realism of recent microfluidic platforms [see Sec. II and Fig. 2(c)], experimentally resolved alveolar and ductal flow patterns are consistent with computational fluid dynamics (CFD) simulations conducted in more realistic space-filling 3D geometries^73,76^ [Fig. 2(d)]. Most recently, microfluidic acinar networks have offered for the first time detailed *in vitro* studies of aerosol deposition patterns under physiological breathing conditions.^41^ Of significance, such measurements have enabled time-resolved imaging of inhaled particle flight trajectories and underlined the complexity of particle dynamics that result from the coupling between gravity, diffusion, and alveolar airflow patterns. These recent efforts have helped pave the path towards direct *in vitro* screening assays of inhaled airborne aerosols in true-scale acinar structures; an encouraging stepping-stone in eventually offering pre-clinical *in vitro* platforms that would circumvent, or at the very least reduce, animal studies.

In some instances, recreating airflows with microfluidics is less pertinent when addressing pulmonary diseases^77,78^ (e.g., mucus hypersecretion in Chronic Obstructive Pulmonary Disease (COPD) and cystic fibrosis) or clinical procedures^79,80^ (e.g., surfactant replacement therapy and mechanical ventilation), where liquid plugs may occlude airways and propagate. This is most relevant in considering the viability of the underlying cellular epithelium (see discussion in Sec. IV) when plug propagation may result in cell damage and injury^81–84^ due to localized, yet severe, flow-induced wall shear stresses (WSS). Microfluidic devices have thus served as potent *in vitro* environments to investigate the dynamics of plug propagation in single airways^81,85^ and bifurcating trees.^82,83,86^ Notably, studies have assessed cellular epithelium injury over the cumulative passage and eventual rupture of the liquid plug;^15^ this latter effect was further underlined in microfluidic alveolar models with the combination of cyclic stretching during breathing that contributes to cell death and detachment.^15,38^

More recently, experiments have also demonstrated that high WSS can disorganize the F-actin cytoskeleton of surfactant-secreting alveolar epithelial cells and thereby limit fusion events of lamellar bodies with the cell membrane prior to exocytosis.^36^ Although these studies have significantly advanced our understanding of plug dynamics, efforts have been mainly limited to Newtonian solutions evocative of surfactant-laden aliquot delivery (i.e., surfactant replacement therapy) to the alveolar regions. There are to date few microfluidic attempts that have concentrated on mucus plug transport.^87^ Indeed, the non-Newtonian, viscoelastic properties of the mucus gel layer^88^ leave open questions on the dynamics of mucus transport and its interactions with the underlying bronchial epithelium, in particular, when subject to rheological changes under bronchial infections or disease^89^ (e.g., cystic fibrosis).

In contrast to life *ex utero*, the developing pulmonary acinar environment during life *in utero* is fluid-filled and subject to spontaneous expansion and contraction motions known as fetal breathing movements.^90,91^ Such movements induce constant cyclic strains recognized to stimulate maturation of the airway epithelium.^92,93^ In particular, alveoli first begin as shallow indentations in the wall parenchyma and gradually evolve during fetal development into more spherical-like cavities over the course of gestational stages.^94,95^ While this configuration has drawn relatively little attention, a recent study in microfluidic models of the developing acinar ducts has revealed a time window during fetal lung life when alveolar flows are anticipated to evolve from attached to recirculating flow structures.^39^ Such efforts represent a first *in vitro* attempt to uncover respiratory flow physiology *in utero* following past efforts limited to numerical simulations.^64–66,96^

## IV. ESTABLISHING BIOLOGICAL FUNCTIONS: THE EPITHELIAL BARRIER AND AIR-LIQUID INTERFACE

The continuous gaseous ventilation occurring throughout life facilitates the exchange of oxygen and carbon dioxide across a delicate architecture known as the alveolar-capillary barrier (ACB). The ACB separates air from blood and may be summarized as constructed of three minimal tissue layers^97^ (Fig. 1, inset): (i) an endothelium lining the capillaries, (ii) an interstitial layer that houses the connective tissue fibres, and (iii) an epithelium lining the airway lumen. While the barrier is very thin (∼1 *μ*m), 75% of all lung cells (by volume and weight) are contained in the gas exchange region^28^ (i.e., lung parenchyma). Importantly, the barrier also regulates the transport of solutes and proteins between capillary blood and alveolar air as well as the clearance of alveolar fluid and liquid homeostasis.^97^ As mentioned earlier (see Sec. II), since the lungs' vast surface area is in direct contact with the external environment, airways are susceptible to microbial infections, injury, and inflammation amongst other; in turn, the ACB also regulates defence mechanisms by controlling the movement of macrophages and lymphocytes from the interstitium and/or capillaries toward the alveolar lumen surface.

The lungs' epithelial cells form an uninterrupted, mosaic-like carpet that continuously lines the lumen of the entire respiratory tract including importantly the gas-exchange region, where the vast majority of lung surface area resides. Although the cellular makeup of the airway epithelium evolves along the respiratory tree from pseudostratified in the upper airways (i.e., ciliated cells, goblet cells, and basal cells) to squamous cells in the deep airway regions, this mosaic of cells establishes consistently a tight monolayer at luminal interface under healthy conditions.^28^ Specifically, in the gas-exchange regions, the epithelium can be identified by two predominant cell types: type I (AEC I) and type II (AEC II) alveolar epithelial cells (Fig. 1, inset). The former are squamous (i.e., flat, scale-like) cells that form the cobblestone-like structure along the alveolar wall. AEC I represent nearly 90% of the alveolar surface^98^ and act thus as the main barrier to foreign aggression.^99^ In contrast, AEC II cells only occupy a minor portion (∼10%) of the entire septal tissue. Yet, these small cuboidal secretory cells accomplish an indispensable role in guaranteeing alveolar functional physiology and autonomous breathing: AEC II are progenitors to AEC I,^100–102^ contribute to innate defence mechanisms^103^ and importantly produce and secrete pulmonary surfactant. Overall, the integrity of the epithelium has been widely shown to correlate with barrier quality.^104,105^ Tight junctional complexes (TJ), i.e., the belt-like proteins that maintain this crucial epithelial continuum, closely bind alveolar cells and create the barrier that essentially limits all translocation across the ACB.^106^ Notably, two of the most characterized alveolar TJ proteins are occludin and zonula occludens (ZO)-1 but a large variety of other TJ complexes characterizes the alveolar architecture, including all epithelial and endothelial TJ proteins.^105^

An additional yet integral component of the defence barrier lies in pulmonary surfactant itself.^103^ This surface-active material is present within the thin liquid lining layer that is formed at the epithelial surface. In combination with air within the lumen, such structure is thus referred to as the air-liquid interface (ALI), as schematically illustrated in Fig. 1 (inset). With the creation of an ALI following birth *ex utero*, surface tension forces at the interface between liquid and gas become dominant and resist distention of the parenchymal walls during breathing. Pulmonary surfactant is thus necessary to mitigate the importance of surface tension along the ALI, such that insufficient concentrations of the surface-active material may render the simple act of breathing excruciatingly difficult. Instability within the parenchyma can then occur, causing alveoli to collapse throughout cycles of inhalation and exhalation. Breathing may be severely compromised, jeopardizing successful oxygen uptake; a condition that arises for example with acute (ARDS) or infant (IRDS) respiratory distress syndrome.^107–109^

Pulmonary surfactant is thus an essential component of the thin liquid lining layer. The latter constitutes an extracellular multiphase film that consists of a thin hypophase covered by the critical surface-active material.^110^ This complex mixture includes various components among which are glycerophospholipids (>90%), with dipalmitoylphosphatidylcholine (DpPC) as the predominant component, and proteins (∼5%) including mainly surfactant-protein (SP)-A, SP-B, SP-C, and SP-D. Additionally, lung surfactant films are known to contribute to innate defence mechanisms. In particular, SP-A, the most abundant surfactant protein, is recognized to play a significant role in the function of airway macrophages.^103^ Morphologically, the aqueous liquid lining layer is acknowledged to be highly dynamic and varies from approximately 10 *μ*m or so in the trachea down to 2.5 *μ*m in the bronchi, reaching finally 0.1–0.2 *μ*m in the peripheral airways^110^ with surface tension values <10 dyn/cm in samples of liquid originating from adult lungs;^111^ such values are in stark contrast with 70 dyn/cm as measured in pure water.^91^ Most astonishingly perhaps, lung surfactant achieves very low, near-zero surface tension values upon film compression during exhalation;^112,113^ a mechanism that is to date still not entirely understood.^114^

Alongside the physical barrier provided by the alveolar epithelium, components of the immune system are imperative to protect the fragile airway complex from external threats. To this end, distal airways accommodate populations of alveolar macrophages and dendritic cells above and beneath the epithelium,^28^ respectively (Fig. 1, inset). While macrophages are the essential phagocytic cell of the alveolar immune system that ingests inhaled foreign material (e.g., particulate matter), dendritic cells act somewhat as a surveillance mechanism, coming into contact with foreign particulate and presenting such detected antigens to other immune cells.^115^

## V. RECREATING AIRWAY BARRIER MODELS *IN VITRO*

The most prevalent approaches^7,17,101,116–119^ acknowledged to recreate models of the airway epithelium originate from (i) animal models, (ii) isolated organs, (iii) tissue explants, and (iv) cell cultures. While animal models have historically been used for over two millennia,^117^ they hold numerous drawbacks, among which their failure to reproduce the intricacies of human diseases. In contrast, externally perfused isolated *ex vivo* human lungs are recognized to mimic most closely the appropriate physiological conditions of human airways *in vivo.*^101,117,118^ However, these latter approaches are often delicate to work with and complex to study. Whether it is the high inter-sample variations, difficulty in interpreting results or challenges in model preparation, isolated *ex vivo* organs are acknowledged to lack the fundamental features required for fast and low cost platforms when addressing, e.g., cytotoxicity and drug screening.^116^ Tissue explants and biopsy samples, on the other hand, offer a simpler alternative; however, their limited life-span remains a shortfall in addition to the 2D nature of the model and the complex procedures for isolation. Moreover, biopsies often harvest insufficient cells whereas explants are frequently obtained from patients with lung diseases.^118^ Overall, *ex vivo* cultures are frequently regarded as offering insufficient advantages over conventional macroscopic cell culture approaches.^116^

Given the aforementioned drawbacks, cell culture models of the airway epithelium have hence been widely used: they enable as a first approximation close examination of the cellular, molecular, and biochemical features of pulmonary diseases^119^ and serve as viable platforms for cytotoxicity and drug screening assays.^120–122^ *In vitro* cell cultures of the pulmonary environment may be distinguished according to the cell origin (e.g., stem cells, primary cells, or cell lines); here, the reader is referred to extensive reviews on the topic^7,8,101,116,118^ Briefly, primary cells are isolated from animal or human lung tissue and exhibit the normal pulmonary phenotype, rendering them a “gold standard” for replicating closely the *in vivo* environment. However, primary cells are rather heterogeneous with limited lifespan; each cell isolation from a subject is unique with high variability between donors or experiments, thus making reproducibility difficult to achieve. In contrast, cell lines are known to be more stable and homogeneous. Yet, their cancerous origin leads to phenotypic differences that render them less ideal for replicating *in vivo* conditions faithfully. We note that most recently, a new cell line, i.e., human Alveolar Epithelial Lentivirus immortalized^123^ (hAELVi), has offered unique properties that are shown to lie between primary cells and immortalized cell lines. The hAELVi cell line has type I-like characteristics with the ability to form tight intercellular junctions, with high trans-epithelial electrical resistance (TEER> 1000 Ω cm^2^). While further detailed characterization is still needed, hAELVis are, however, the first cell line demonstrating barrier properties of the alveolar epithelium.

**FIG. 4.**
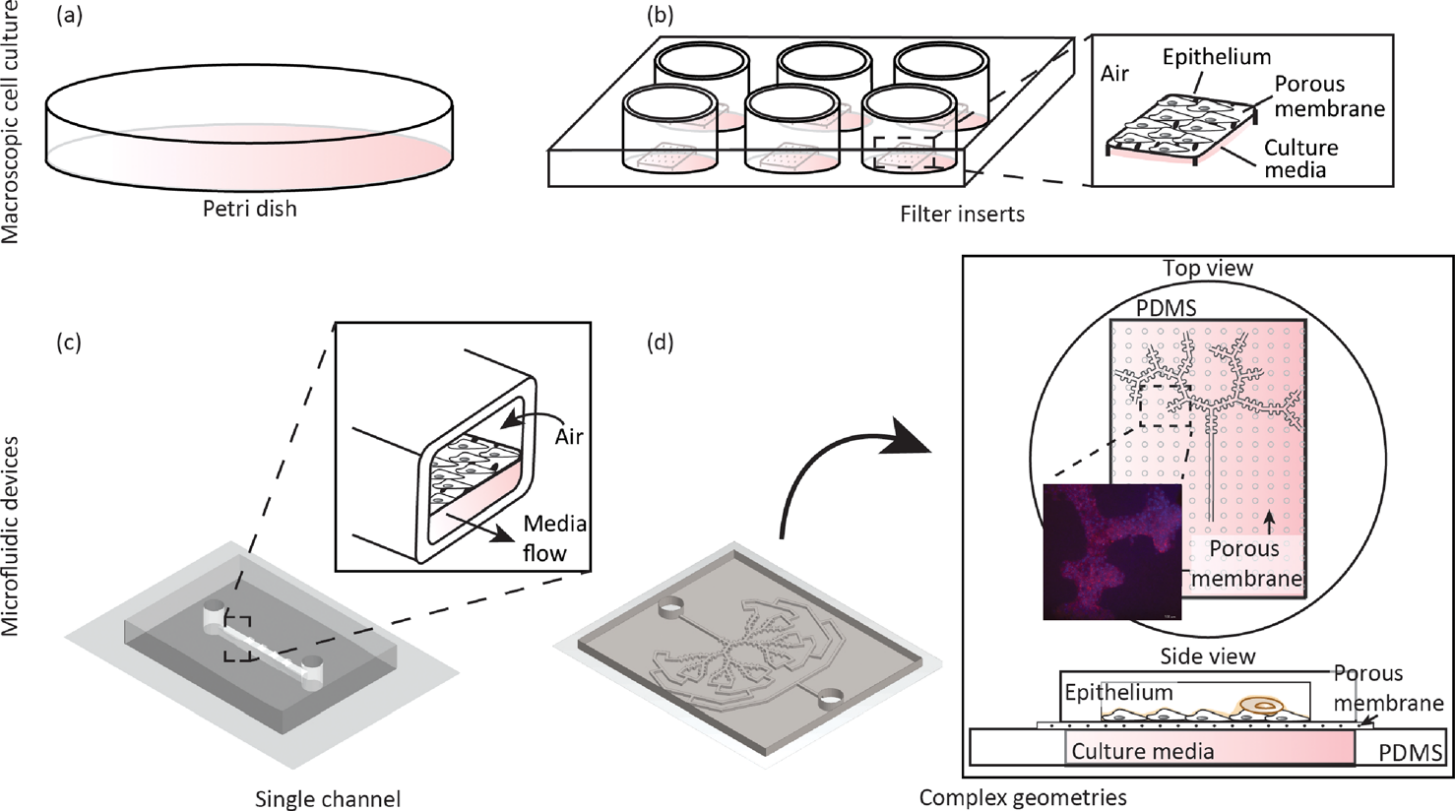
Schematic evolution of *in vitro* cellular platforms of the pulmonary environment recreating an airway epithelium (e.g., bronchial or alveolar, cell lines or primary cells, etc.). Traditional macroscopic cultures have revolved around (a) a petri dish to culture cells under immersed conditions in culture media, falling short of realizing an air-liquid interface (ALI). More faithful macroscopic approaches have followed including (b) the use of filter inserts inside multi-well plates, thereby recreating an ALI by exposing the apical side of the cells to air (see inset). With microfluidics, (c) pulmonary cell cultures inside airway analogues have been made possible with seminal designs such as a single channel featuring a porous membrane separating an air-exposed apical side representing a simplified model of an ALI, where cells are cultured and a basal side where media is flown (see inset). In the footsteps of seminal single airway ALI models, an embodiment of an anatomically inspired, multi-generation airway tree platform is shown in (d) with a A549 cell line seeded on the apical side of a porous membrane (see inset) and a pool of culture media that is perfused from the basal side; cells were allowed to grow for 5 days under immersed conditions followed by an additional 5 days at an ALI. Inset: fixed cells where stained with Phalloidin (actin staining, red) and DAPI staining (nuclei, blue).

Whether operating with primary cells of alternatively cell lines, the most prevalent method for cell culturing consists of a macroscopic 2D sheet made of epithelial or “epithelial like” cell types and grown on specialized dishes [Fig. 4(a)]. In general, cell cultures grown in petri dishes enable fast and low cost studies that identify on a cellular level, morphological, physiological, metabolic, and genetic responses of particular cells to new developments of drugs or toxicants.^117,124,125^ Undeniably, an oversimplified model such as a 2D cell sheet cannot capture many key aspects of the airway complexity, including anatomical (see Sec. II) and physiological flow (see Sec. III) considerations. In particular, cell cultures are acknowledged to lead to misinterpretations and false conclusions.^3^ A possible strategy to reconstitute more authentically the innate cellular makeup stems in integrating several cell types within the cell culture,^126,127^ i.e., co- or triple-cultures of the airway epithelium in conjunction with other cell types (e.g., immune cells). Yet, with increasing biological complexity, the robustness of cell culture assays may decline, resulting in higher variability and less predictable results.^3^

In this context, cell cultures grown on permeable microporous membranes [Fig. 4(b)] represent an important step forward in reconstituting a viable ALI *in vitro*. Such setups mimic more faithfully the intrinsic epithelial structure where the topical side of the membrane is exposed to air, granting the cells its polarized nature and recreating the essentials of the ALI. Alternatively, other specialized culturing methods have strived to innovatively integrate dynamics of the liquid lining during breathing at the ALI. This includes, for example, a technique based on captive bubble surfactometry;^128^ a system that cyclically compresses the surfactant film covering an air-exposed epithelial monolayer. This latter approach has helped to suggest an influence of surface tension dynamics (as described in Sec. IV) on particle-cell interactions. Both air-exposed methods (i.e., permeable membranes and surfactometry) successfully establish a two-chamber system that enables the exposure of cells to air on the apical side while providing media support on the basal side of the cultured monolayer. As such methods attempt to portray a more genuine *in vivo* environment, air-exposed culture conditions ultimately change the resulting cellular phenotype. While cell cultures grown in petri dishes have been traditionally conducted under immersed conditions, those at an ALI are known to increase monolayer integrity^120,123,126,129^ (measured by trans-epithelial electrical resistance, TEER), contribute to well differentiated morphology in primary cells,^130,131^ and increase surfactant secretion for AEC II cells.^126,129^ Hence, culture methods at an ALI have been increasingly attractive over the past years,^118,132^ emphasizing such method as a favourable platform to investigate the epithelial barrier at large^126,129^ as well as cytotoxicity in the context of inhaled xenobiotics^132–136^ and drug transport.^137–139^

Similar to the classic cell cultures in dishes, integrating co-cultures of different cell types on permeable membranes [Fig. 4(b)] has increased the complexity of oversimplified models of the airway epithelium and thereby mimic more closely the ALI *in vivo*. For example, immune-components have been integrated within the airway epithelium, including alveolar macrophages and dendritic cells.^115,126,131,140,141^ When considering cell-particle interactions within ALI-based culture systems, particle uptake by the epithelium has been shown to differ between mono-cellular and co-cultures with integrated immune cells, showing preferential uptake of particles by the immunological cells relative to the epithelial monolayers.^126,129,142^ Most recently, 3D printing techniques have enabled to reconstruct in an automated and reproducible fashion an *in vitro* ACB composed of endothelial cells, a basement membrane, and epithelial cells.^143^ Such advancements circumvent traditional manual methods with the creation of thinner and more homogeneous cell layers that are required for an optimal air-blood tissue barrier.

## VI. MICROFLUIDIC PLATFORMS OF THE PULMONARY ENVIRONMENT

The biological intricacies of the pulmonary environment represent an undeniable challenge to accurately recreate *in vitro*. In this context, the bulk of existing efforts still fall short in mimicking sufficiently adequately the airway milieu, both in a physiologically faithful manner and at true physiological scale. Notably, macroscale techniques do not capture the intricate 3D airway anatomy, as described earlier in Sec. II, in particular, when considering small airways and the gas-exchange region. Furthermore, *in vitro* cell cultures are still inherently confined to static culture conditions and largely omit respiratory airflows and the dynamic nature of the airway epithelium during breathing, as detailed earlier in Sec. III.

With the advent of microfluidics and the birth of *lung-on-chips*, several limitations surrounding traditional macroscopic culture methods (i.e., petri dishes, permeable membranes, etc.) have been tackled if not addressed with increasing success. As a brief remark, we comment here that *lung-on-chip* micro-devices have been instrumental in helping the general field of *organ-on-chips* evolve.^1,3,26^ Many of the early *lung-on-chip* platforms first appeared during the development of *in vitro* models of liquid plug propagation in an effort to explore the effect of such plugs on the underlying epithelium (see Sec. III). These microfluidic systems were among the first to miniaturize the airway environment to true scale and integrate a viable ALI at the surface of a confluent epithelium.^15,144^ Though originally limited to a single airway channel and a single cell line (e.g., alveolar Type II epithelia), these microdevices set a precedent in capturing biological functionality at length scales directly comparable to the innate pulmonary environment. Importantly, such microfluidic setups have helped deliver cellular *in vitro* models cultured directly in airway channels (rather than plates or inserts) and thereby replicate more faithfully physiological fluid shear forces (i.e., WSS) and solid mechanical forces (i.e., stretching) anticipated to act on the underlying lung epithelium. Together, this helped consolidate a departure from more traditional macroscopic approaches.

Undoubtedly, the work of Huh *et al.*,^23^ reviewed on several occasions in the context of *organ-on-chips*,^2,26^ represents a significant leap forward in advancing microfluidic designs of the pulmonary environment at a suitable microscale and successfully integrating cyclic stretching of the ACB to mimic physiological breathing strains. Briefly, the original platform revolved around a straight microchannel designed as a two-chamber structure [Fig. 4(c)] that integrates a co-culture of (type II alveolar) epithelial and endothelial monolayers cultured on opposing sides of a porous PDMS-based membrane. The microfluidic device allowed to directly observe and quantitatively analyse physiological functions of the ACB and provided a platform to test cytotoxicity effects, specifically of silica nanoparticles, i.e., a widely used particle model to assess the toxicity of ultrafine aerosols. Amongst other, their results showed that stretching of the sandwiched thin porous membrane under realistic strains plays a dominant role in enhancing nanoparticle transport through the ACB. Additionally, the device was further used to recapitulate an inflammation cascade at the ACB where flowing white blood cells (WBC) were shown to transmigrate from the endothelial-lined vascular compartment into the epithelial side and thereafter engulf bacteria.

Other notable microfluidic designs under air-exposed ALI conditions have followed, with a keen eye on translational research and therapeutic applications. Such efforts have principally focused on airway disease models such as a micro-engineered ACB platform for pulmonary edema,^16^ pulmonary thrombosis,^20^ and a small *airway-on-a-chip* model for COPD studies.^24^ The former study utilizes the same device and alveolar epithelium (i.e., type II A549 cell line) as in Huh *et al.*^23^ to investigate barrier integrity as a result of cytotoxicity arising from therapeutics (e.g., chemotherapy), showing that stretching of the epithelium compromises the pulmonary barrier. Additionally, the same device was leveraged to test a new pharmacological agent (i.e., GSK2193874) that showed to inhibit leakage and oedema. The latter *airway-on-a-chip* considers instead a bronchial epithelium representative of small airways susceptible to COPD. Culture of primary cells in such device produced ciliated epithelial cells as well as mucus-secreting goblet cells, club cells, and basal cells in proportions similar to those found in normal human lungs. Using COPD patient cells, important features of the disease were recapitulated including selective cytokine secretion, neutrophil recruitment, and exacerbation triggered by an infection.

Most recently, Benam *et al.*^18^ have taken such disease modelling a step forward by exposing a patient-specific small breathing airway chip to smoke arising from regular and electronic cigarettes, allowing for the monitoring of the smoke-induced functional changes *in vitro*. Here, the airway platform (i.e., geometry air volume and shear stress) was designed to mimic mid- to small-bronchi (i.e., generations 8–16 of the lungs). In order to identify the effects of smoking on healthy and COPD donors, the authors coupled the organ-on-chip device, seeded with a human bronchiole epithelium from either of the two groups, to a smoke generator and a micro-respirator to mimic human breathing behaviour. Briefly, the micro-respirator is programmed to breathe cyclically microliter volumes of air through the device's upper epithelium-lined channel and a smoke machine regulates smoking parameters (e.g., puff duration and volume, inter-puff interval, puffs per cigarette). As the micro-respiratory and smoke generator work synchronously, whole cigarette smoke flows horizontally across the surface of the differentiated epithelium only during the inhalation phase of the respiration cycle and then flows back out during the exhalation phase. Upon inspection of the gene expression profiles of COPD samples exposed to smoke, the authors identified 276 genes differentially expressed compared to control samples without smoke, of which 147 genes were previously associated with COPD pathogenesis. Using real-time imaging, ciliary beating was monitored and changes as a result of smoke exposure were assessed. Overall, this work exemplifies important advantages of organ-on-chip platforms over more traditional alternatives devoid of real-time monitoring. Such efforts are also anticipated to help pave the way in discovering new therapeutic drug targets and more customized treatments.

In parallel to these studies, innovations in microfabrication techniques themselves have led to reach out beyond the use of simple (rectangular) channels. For instance, a recent micro-device recreating the dynamic nature of an ALI has integrated a bio-inspired diaphragm-like actuation mechanism to mimic 3D cyclic strains that act upon epithelial layers.^145^ Other examples have offered instead a synthesis between microporous membrane support and a microfluidic perfusion system to enhance the air-interfaced cell culture. These include notably (i) an alveolar airway model to investigate epithelium viability and monolayer integrity at the ALI,^146^ (ii) a bronchial model that offers temporal analysis of the cellular response following exposure of cultured epithelium layers to environmental particulates,^147^ (iii) human airway models that display mucociliary differentiation and barrier function using solely primary cells cultured in vertically stacked, individually accessible compartments separated by membranes,^148^ (iv) bronchial and tracheal models that scrutinize the effect of airflow-induced shear stress on epithelial layer organization, mucin secretion, liquid absorption, and barrier function,^149^ and finally (v) nasal models to investigate hazardous effects of toxicant exposure on nasal epithelium cells.^150^ Beyond the pulmonary domain, the prospect of air-interfaced cell cultures as demonstrated by the growing list of *lung-on-chip* models has inspired the design and development of other organ-specific microfluidic platforms such as the *gut-on-chip*;^151^ a biomimetic model of yet another biological microenvironment that experiences contact of a cellular barrier directly with air and expresses a polarized epithelium.

**FIG. 5.**
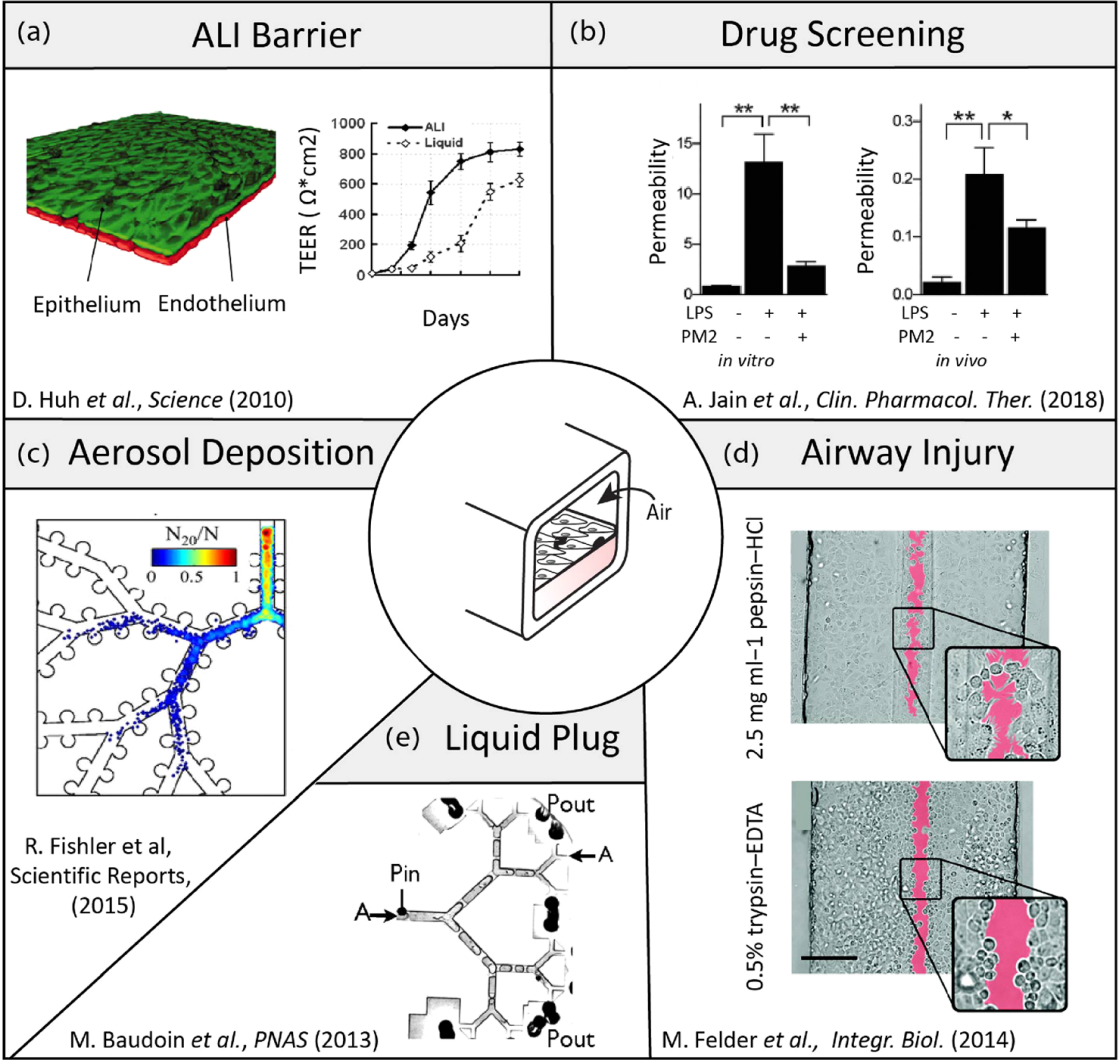
Examples of research efforts across areas of respiratory physiology advanced and supported by microfluidic *airway-* and *organ-on-chip* platforms. (a) The seminal work of Huh *et al.* ^23^ demonstrates a biomimetic reconstitution of a functional alveolar-capillary barrier (ACB). There, a tissue-tissue like interface was designed, where a single layer of the alveolar epithelium was seeded on one side (apical) of a porous PDMS membrane while on the other (basal) side a monolayer of endothelium is grown. The barrier properties were characterized by staining of the intercellular junctional structures and TEER measurements. Reproduced with permission from Science **328**, 5986 (2010).^157^ Copyright 2010 American Association for the Advancement of Science. (b) Jain *et al.*^20^ demonstrated that a microfluidic *alveolus-on-a-chip* lined with both human primary alveolar epithelial and endothelial cells cultured under whole blood perfusion can be leveraged to identify antithrombotic therapeutics. Reproduced with permission from Clin. Pharmacol. Ther. **103**, 2 (2018).^158^ Copyright 2018 Wiley-Blackwell. (c) Fishler *et al.*^41^ explored the fate of inhaled aerosols in a true-scale microfluidic acinar airway tree, undergoing oscillatory wall motion during breathing and imaged resulting particle deposition patterns. Reproduced with permission from Sci. Rep. **5**, 14071 (2015). Copyright 2015 Nature Publishing Group. (d) Felder *et al.*^84^ present a microfluidic lung epithelial platform that allows for the selective exposure of alveolar epithelial cells to gastric contents and explore ensuing epithelial wounding phenomena. Reproduced with permission from Integr. Biol. **6**, 12 (2014).^159^ Copyright 2014 Royal Society of Chemistry. (e) Baudoin *et al.*^86^ investigated in a multi-generation bifurcating airway tree the dynamics of liquid plug ruptures across the network. Reproduced with permission from Proc. Natl. Acad. Sci. U.S.A. **110**, 859 (2013).^86^ Copyright 2013 National Academy of Sciences.

**FIG. 6.**
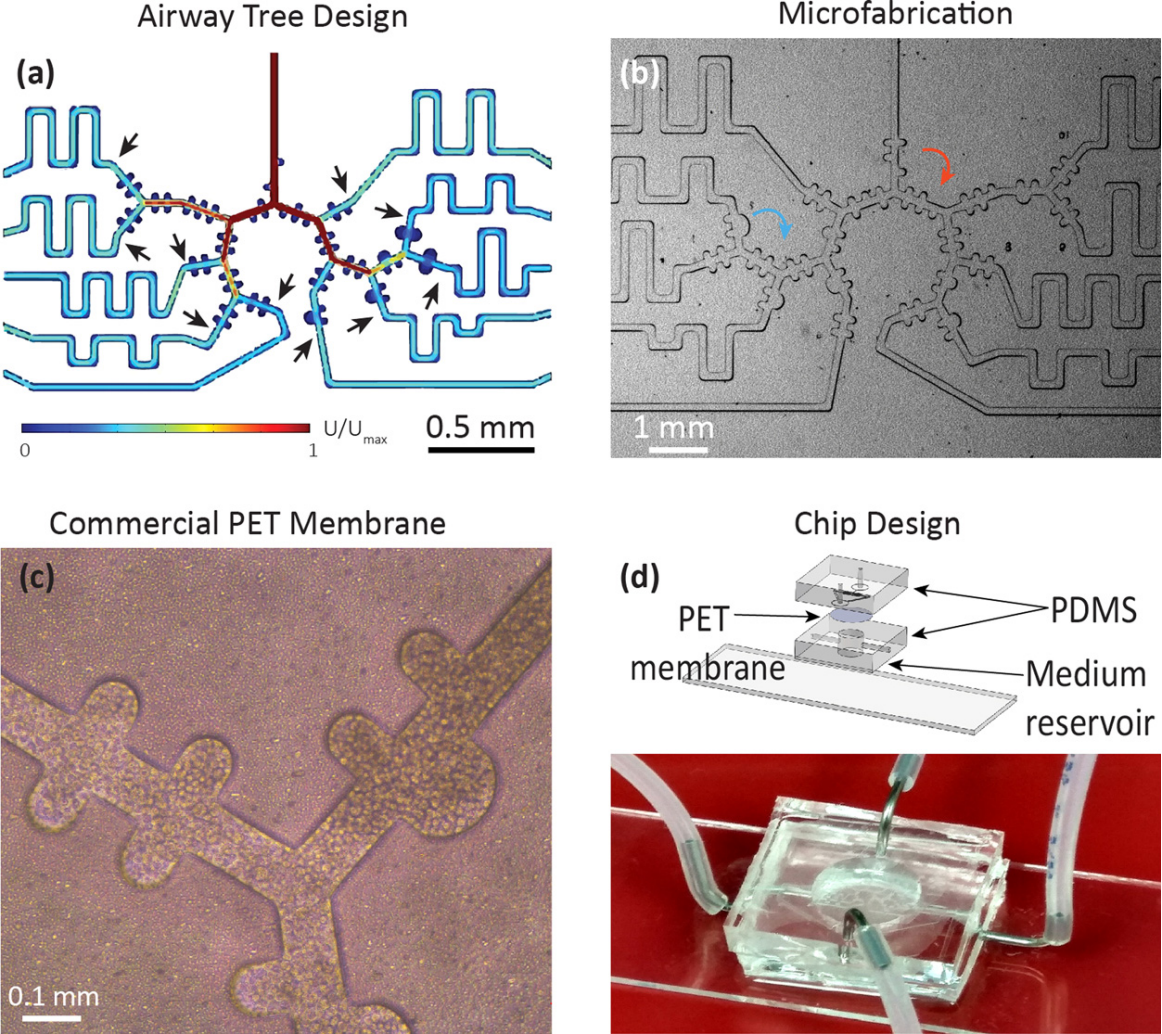
Example of a microfluidic alveolated airway tree design featuring a multi-generation asymmetrically bifurcating model of a sub-acinar structure modelling (early childhood) airways. (a) As one departs from simple isolated airway channels, computational fluid dynamics (CFD) simulations are often sought in the design and layout of the microfluidic pattern. Here, the microfluidic device is designed to provide equal flow to each terminal end of the model (i.e., flow resistance matching) by adjusting the length of a channel connecting the common microfluidic outlet and the last generation of the acinar tree (arrows) such that equal flow at each terminal end of the acinar model is guaranteed. Colour coding corresponds to velocity magnitudes where U_max_ is the maximal flow at the entrance of the domain under steady-state conditions. (b) Corresponding microfluidic PDMS-based model of developing acinar airways, demonstrating saccular alveolar spaces (blue arrow) and under-alveolated acinar ducts (red arrows). Close-up of the same microfluidic tree structure using (c) close-up of a commercial PET membrane (DOW Corning) (10 *μ*m thick) with 3 *μ*m pore size, seeded with A549 cell line. (d) Upper panel: Exploded and assembled computer-aided drawing (CAD) views of a pulmonary tree-on-a-chip device [similar to schematic of Fig. 4(d)]. A compartmentalized sandwich structure is assembled on a glass slide. A porous membrane is positioned between a PDMS airway tree (apical side) and a reservoir (bottom side) to perfuse culture media in the basal side (with openings for inlet and outlet). On the apical side, two openings (inlet and outlet) allowing selective insertion of desired components to the upper tree, e.g., cells for seeding followed by airflow to reproduce physiology flow conditions. Bottom panel: View of a complete pulmonary tree-on-a-chip device.

Overall, astute designs of microdevices featuring compartmentalized architectures^15,16,152^ and moving walls^23,151^ have been instrumental in shedding new light on physiological and biological questions revolving around barrier integrity and function, particle translocation, cytotoxicity, dosimetry, and inflammatory responses amongst other. This is exemplified in the broad range of pulmonary-related research currently explored *in vitro* with the support of microfluidic platforms (Fig. 5): notable efforts in the above-mentioned areas include amongst other (i) exploring ALI and ACB barrier characteristics,^23^ (ii) conducting therapeutic drug screens,^20^ (iii) mapping aerosol deposition patterns in airways,^41^ (iv) quantifying the dynamics of liquid plug transport,^86^ and (v) assessing airway epithelial injury.^84^ In further advancing the field to deliver respiratory platforms that meet the complex integration of anatomical, physiological, and biological constraints, microfluidic designs must strive to recreate more faithfully whole-organ functions; a condition that would encourage expanding the breadth (i.e., scaling-up) of microfluidic platforms [e.g., Figs. 6(a) and 6(b)] and encompassing the diversity of the pulmonary cellular makeup within (i.e., co- and triple cultures, immune cells, etc.). While many of the more prominent designs now revolve around the use of either custom-designed^5,23,151^ or commercially available^153^ [Fig. 6(c)] thin porous membranes that are integrated within multi-layered PDMS-based chips [Fig. 6(d)], it must be emphasized that these attractive techniques remain at best a proxy for the innate septal barrier exhibiting limited biological functionality. We recall that *in vivo* the air-blood barrier has a mere ∼1.6 *μ*m thickness, composed of a single basement membrane coated by two cytoplasmic lamellae (from the endothelial and epithelial cells), whereas microporous membranes are typically on the order of 10 *μ*m thick and thus cannot mimic the same translocation and diffusive properties. In turn, such artificial membranes are still a far reach from replicating the complex tissue barrier (e.g., cellular make-up, extracellular matrix, dense capillary networks embedded, etc.) and its associated *in vivo* transport characteristics (e.g., diffusion, translocation). Nevertheless, the use of artificial porous membrane structures has been essential in advancing our general understanding of pulmonary physiology, notably in the areas of ALI barrier characteristics, drug translocation, and airway injury amongst other (Fig. 5).

In observing the current state-of-the-art, microfluidic platforms are still inherently limited in mimicking just a few of the aforementioned requirements and long-term goals despite impressive progress achieved in little more than a decade. This is perhaps best exemplified by observing that on the one hand the most faithful *in vitro* epithelial barrier models of the ALI are still confined to single, isolated airway micro-structures [Fig. 4(c)] with moderate realism in airway morphology and the respiratory flows reproduced thereby, whereas the most advanced breathing airway tree micro-structures devised to date (Figs. 5 and 6) still remain largely restricted to physical models in the absence of extensive biological functionality. In an attempt to look beyond such hurdles, one potential source of inspiration may stem from the field of tissue engineering where considerable progress over the past two decades has allowed, for example, the transplantation of *in vitro* growth tissues and organs in humans. Indeed, a diversity of scaffold designs has been developed and grown in laboratories, lined with patients' specific cells to avoid rejection by the immune system. While the end-goals of such endeavours may differ from the *in vitro* airway platforms discussed and sought here (e.g., the transplantation of tissues such as blood vessels^154^ and dermis^155^), the bioengineering approaches put forth are nevertheless noteworthy. In particular, bioprinting and cell-laden hydrogels^143^ represent emerging techniques that enable the fabrication of more physiologically relevant, complex 3D structures. We raise here as a final note the argument that, thus far, the potential for such technologies has not yet been fully explored or leveraged by the *organ-on-chip* community, in the hope that their integration will foreseeably open new possibilities.

## VII. CONCLUSIONS

With formidable progress undergone in miniaturization processes and microfabrication techniques, microfluidic analogues of the pulmonary environment have already transformed the landscape for exploring *in vitro* the breadth of questions underpinning respiratory physiology. While numerous hurdles still stand in the way towards integrating cohesively anatomical, physiological, and cellular aspects into a faithful *in vitro* respiratory organ, microfluidic-based strategies have nevertheless provided profound new opportunities to probe at true scale the pulmonary environment and deliver ever closer biomimetic platforms of the innate *in vivo* milieu, even if still limited in biological functionality.

**FIG. 7.**
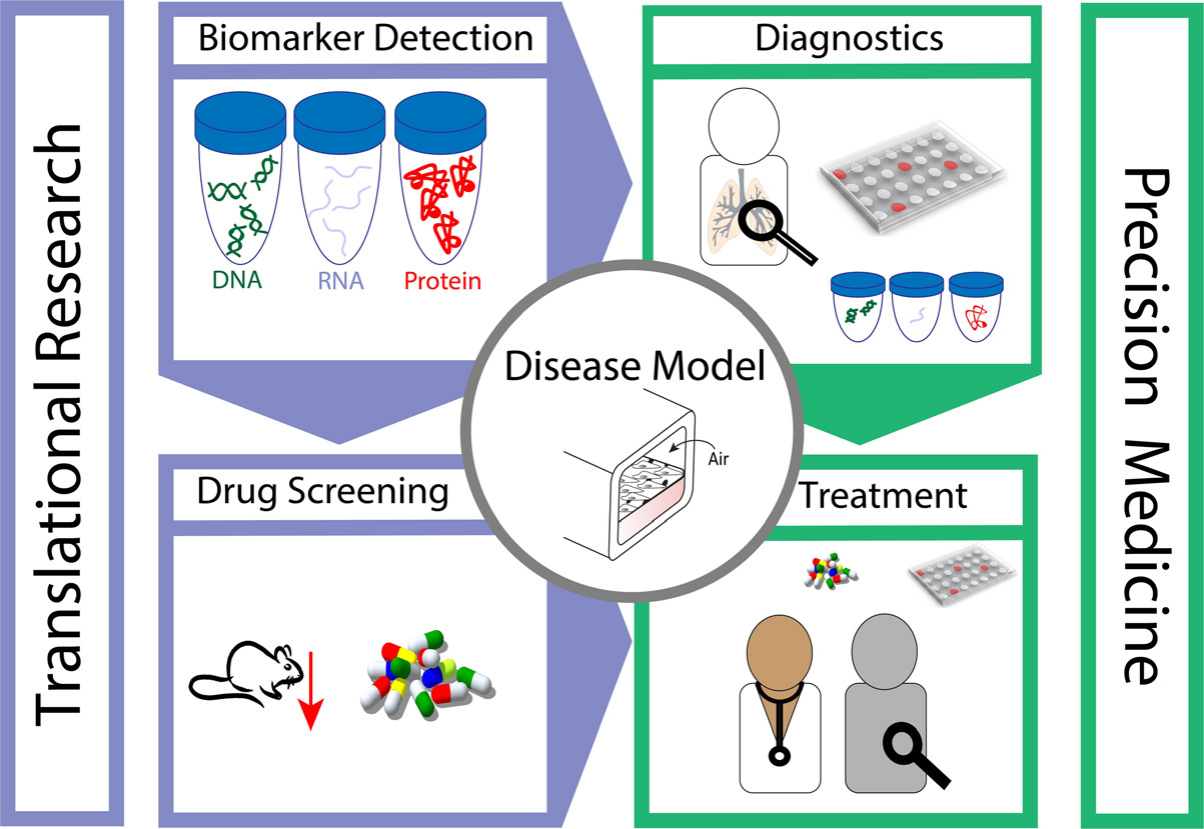
Schematic embodiment of future directions. Lung-on-chip platforms hold the potential to advance applications ranging from identifications of new biomarkers, at all levels (e.g., proteins, DNA, and RNA) up to drug screening, and reducing animal testing, with the aim to advance the understanding of disease mechanisms. As the platform permits *in vitro* examination of devices lined with a patient's own cells, the gained knowledge can offer new physiological insights, in both health and disease, complimentary to current tools available for diagnostics. Moreover, chips offer the prospect of matching between a patient's specific needs and available therapeutics, thereby advancing the field of precision medicine.

With an outlook on prospective directions, the schematic of Fig. 7 embodies a summary of possible future applications; *organ-on-chip* platforms have the potential to advance both basic research as well as translational medicine. Notably, developing disease modelling on chip is anticipated to foster the discovery of new biomarkers by exposing *in vitro* chips to diverse conditions and monitoring various cellular responses, e.g., from cell morphology, using real-time imaging microscopy, to changes in proteins expression, and DNA\RNA regulations changes, experiments that are still beyond reach *in vivo*. The discovery of new biomarkers may hence deliver new insight on diseases mechanisms as well as offer new targets for drug developing. Since drug screening methods are still overwhelmingly conducted in animal models, *organ-on-chips* offer the prospect of tangible alternatives. Lined with human cells, within a physiologically faithful biomimetic architecture, these can help not only reduce the need for animal studies but also offer in some cases improved and more relevant (human) models. Integrating the above is anticipated to open the door for advanced precision medicine: patients' cells can be collected from a biopsy, expanded, and grown inside devices allowing advanced diagnostic capabilities in addition to monitoring patient's cell response to different drugs for optimizing match.

It remains still largely speculative whether *lung-on-chips* and other similar micro-devices will live up to the promise of becoming an accepted gold standard for various testing and screening assays, and thus fulfilling many if not most of the discussed aims herein. Even if such realizations are not necessarily met, the rapid progress witnessed in a single decade has already transformed our perception of *in vitro* macroscale approaches and underlined our criticism thereof, with an increasing urge to move beyond such traditional techniques.
